# Supplementary material for: Effect of Gardeniae Fructus Powder on Growth Performance, Antioxidant Capacity, Intestinal Barrier Function, and Colonic Microbiota of Weaned Piglets
Source: Animals (Basel). 2025 Jan 15;15(2):221. doi: 10.3390/ani15020221 (PMC11758313; doi:10.3390/ani15020221)
Supplement: Supplementary file 1 [file animals-15-00221-s001.zip › animals-3392100-supplementary.pdf]

Attachment

Supplementary Table S1. The nutritional levels and main bioactive compounds of GF powder.

Items	Content
Crude protein	10.21%
Crude fat	16.60%
Crude fiber	23.20%
Crude ash	3.70%
Calcium	0.24%
Total phosphorus	0.17%
Total flavonoids	3.23 mg/g
Total phenol	1.2 μmol/g
Soluble sugar	6.50 mg/g
